# Early diagnosis of retinal neurovascular injury in diabetic patients without retinopathy by quantitative analysis of OCT and OCTA

**DOI:** 10.1007/s00592-023-02086-z

**Published:** 2023-05-05

**Authors:** Baohua Li, Wenwen Li, Chaohong Guo, Chengwei Guo, Meirong Chen

**Affiliations:** 1 The First Clinical Medical College, Shandong University of Traditional Chinese Medicine, No. 4655 Da-Xue Road, Jinan, 250355 Shandong Province People’s Republic of China; 2 Ophthalmology Department of Shandong Hospital of Traditional Chinese Medicine, No. 16369 Jing-Shi Road, Jinan, 250013 Shandong Province People’s Republic of China

**Keywords:** Early diabetic retinopathy, Diagnostic capacity, OCT, OCTA, Retinal thickness

## Abstract

**Aims:**

To quantitatively analyze and compare the differences in retinal neurovascular units (NVUs) between healthy individuals and patients with type 2 diabetes mellitus (DM) by optical coherence tomography (OCT) and optical coherence tomography angiography (OCTA) techniques and to determine the value of this technique for the early diagnosis of retinal neurovascular damage in patients with diabetes mellitus without retinopathy (NDR).

**Methods:**

This observational case‒control study was conducted from July 1, 2022, to November 30, 2022, at the outpatient ophthalmology clinic of the Affiliated Hospital of Shandong University of Traditional Chinese Medicine. All subjects underwent baseline data entry and mean thickness of the peripapillary retinal nerve fiber layer (pRNFL), the thickness of each retinal layer in the macula 3 × 3 mm, and vascular density (VD) examination.

**Results:**

The study included 35 healthy individuals and 48 patients with DM. The retinal VD as well as partial pRNFL, macular nerve fiber layer (NFL), and macular ganglion cell layer (GCL) thickness in DM patients exhibited significantly lower VD in the DM group than in the control group (p < 0.05). Age and disease duration of DM patients showed a negative trend with pRNFL thickness, macular NFL thickness, macular GCL thickness, and VD. However, a positive trend was observed between DM duration and partial inner nuclear layer (INL) thickness. Moreover, there was a positive correlation between macular NFL and GCL thickness and VD for the most part, while a negative correlation was shown between INL temporal thickness and DVC-VD. pRNFL-TI and GCL-superior thickness were screened as two variables in the analysis of the predictors of retinal damage in DM according to the presence or absence of DM. The AUCs were 0.765 and 0.673, respectively. By combining the two indicators for diagnosis, the model predicted prognosis with an AUC of 0.831. In the analysis of retinal damage indicators associated with the duration of DM, after regression logistic analysis according to the duration of DM within 5 years and more than 5 years, the model incorporated two indicators, DVC-VD and pRNFL-N thickness, and the AUCs were 0.764 and 0.852, respectively. Combining the two indicators for diagnosis, the AUC reached 0.925.

**Conclusions:**

Retinal NVU may have been compromised in patients with DM without retinopathy. Basic clinical information and rapid noninvasive OCT and OCTA techniques are useful for the quantitative assessment of retinal NVU prognosis in patients with DM without retinopathy.

## Introduction

The prevalence of diabetes mellitus (DM), a serious, long-term disease, continues to rise rapidly worldwide [1]. According to the International Diabetes Federation, there are nearly 500 million people with DM worldwide, and 579 million people are expected to have DM in 2030, a number that will increase to 700 million in 2045 [2]. Diabetic retinopathy (DR), a major complication of DM, occurs in approximately 30%–40% of diabetic patients. Globally, more than 100 million people live with DR, and it is the leading cause of vision loss in middle-aged and older adults [3, 4]. DR has been considered a microvascular disease. The earliest response of the retinal vasculature to hyperglycemia is vasodilation and altered blood flow, triggering pericyte loss, endothelial cell apoptosis, and basement membrane thickening [5, 6]. Clinical manifestations such as retinal hemorrhages and microaneurysms are present when the damage to the retina is irreversible. Recent studies have shown that retinal neurodegeneration may precede microaneurysms [7]. Therefore, the understanding of DR has shifted from simple microvascular injury to the imbalance of neurovascular unit (NVU) injury and its coupling mechanism [8]. The structure of the NVU consists of retinal neurons, glial cells, and the retinal microvascular system, which are distributed in the corresponding 10 layers of the retina. Early detection and diagnosis have become the goals of many researchers, and because of its insidious onset, there is an urgent need for improved means of monitoring the progression of DR.

For many years, fluorescein angiography (FA) has been used as the reference method for visualizing retinal circulation. This 2D invasive technique, in addition to the potential risk of intravenous injection, does not allow for precise stratification of the retina and imaging of the blood vessels specifically around the optic disk [9, 10]. In recent years, the introduction of optical coherence tomography (OCT) has revolutionized the visualization and quantification of retinal stratification [10]. Optical coherence tomography angiography (OCTA) is a new noninvasive imaging modality that allows visualization of the microvasculature of the retina and choroid in different layers, allowing easy measurement of microvascular changes in the peripapillary and macular regions [11]. This technique allows easy and accurate measurement of retinal changes in diabetic patients without DR. Previous studies have mostly focused on microcirculation in the macula or optic disk area or on changes in the thickness of the retinal nerve fiber layer (NFL) [12–14]. In contrast, studies focusing on the NVU using OCT or OCTA techniques are limited. We know that the NVU is mainly distributed in the retinal NFL to the inner nuclear layer (INL). The quantitative analysis of the thickness of the main distribution levels of the NVU in the retina and blood flow densitometry allow a comprehensive assessment of the imbalance of the coupling mechanism of the retinal NVU in diabetic patients without DR.

In this observational case‒control study, we quantified OCT and OCTA indices of the optic disk and macula in type 2 diabetic patients without DR and healthy subjects, respectively, to assess the interconnection between retinal nerve, glial cell, and microvascular damage in early diabetic patients and to provide diagnostic value for the prevention of DR.

## Methods

### Subjects

This study was approved by the Ethics Committee of the Affiliated Hospital of Shandong University of Traditional Chinese Medicine, Approval No. (2021) Ethics Audit No. (013)-XY, in accordance with the Declaration of Helsinki guidelines. Informed consent was obtained from all participants. This cross-sectional single-center study involved 48 patients diagnosed with type 2 diabetes using standard methods and 35 healthy individuals as controls. The participants underwent routine ophthalmologic examinations, including best-corrected visual acuity (BCVA), slit lamp microscopy, direct ophthalmoscopy, and noncontact tonometer for intraocular pressure (intraocular pressure (IOP). Age, sex, highest fasting blood glucose concentration, and duration of diabetes were also recorded. The BCVA was checked using the international standard visual acuity chart, which was converted into logarithm of the minimal angle of resolution (logMAR) visual acuity for statistical analysis. Two independent investigators (BHL and WWL) reviewed all medical records and fundus conditions to determine the status of diabetic retinopathy and excluded patients with abnormal fundus findings associated with diabetic retinopathy, such as retinal hemorrhages, exudates, and cotton wool spots.

Inclusion criteria: Age ≥ 18 years; IOP ≤ 21 mmHg; spherical equivalent (SE) between + 3.00 (diopters) D and -3.00 D. Met the definition of type 2 diabetes mellitus with a randomized blood glucose concentration of ≥ 200 mg/dL (11.1 mmol/L) according to the criteria recommended by the World Health Organization dL (11.1 mmol/L) or a fasting glucose concentration of ≥ 126 mg/dL (7.0 mmol/L) or a plasma glucose concentration ≥ 200 mg/dL (11.1 mmol/L) 2 h after a 75 g oral glucose load [15].

Exclusion criteria: diagnosed diabetic retinopathy, high myopia, glaucoma, anterior retina, hypertensive retinopathy, macular degeneration, retinal hemorrhage, retinal vascular obstruction or uveitis; intraocular surgery such as vitrectomy, cataract surgery, glaucoma surgery, etc.; diseases that affect the quality of OCT and OCTA scans, such as nystagmus, ulcerative keratitis, cataract, etc.

### OCT and OCTA

All subjects underwent OCT (Spectralis, Heidelberg Engineering Gmbh, Germany) for corresponding measurements of the macula and optic disk area, with images taken in natural light. Patients were seated, and all subjects were fully dilated with 0.5% tropicamide before the examination, with the lower jaw placed in the jaw rest and the forehead against the frontal rest. The height of the jaw rest was adjusted, and the patient was instructed to look inwardly at the visual standard. All scans were performed by the same experienced examiner (LWW), and all scans were reviewed individually by two researchers (HBL and CMR).

### pRNFL thickness measurement

**Fig. 1:**
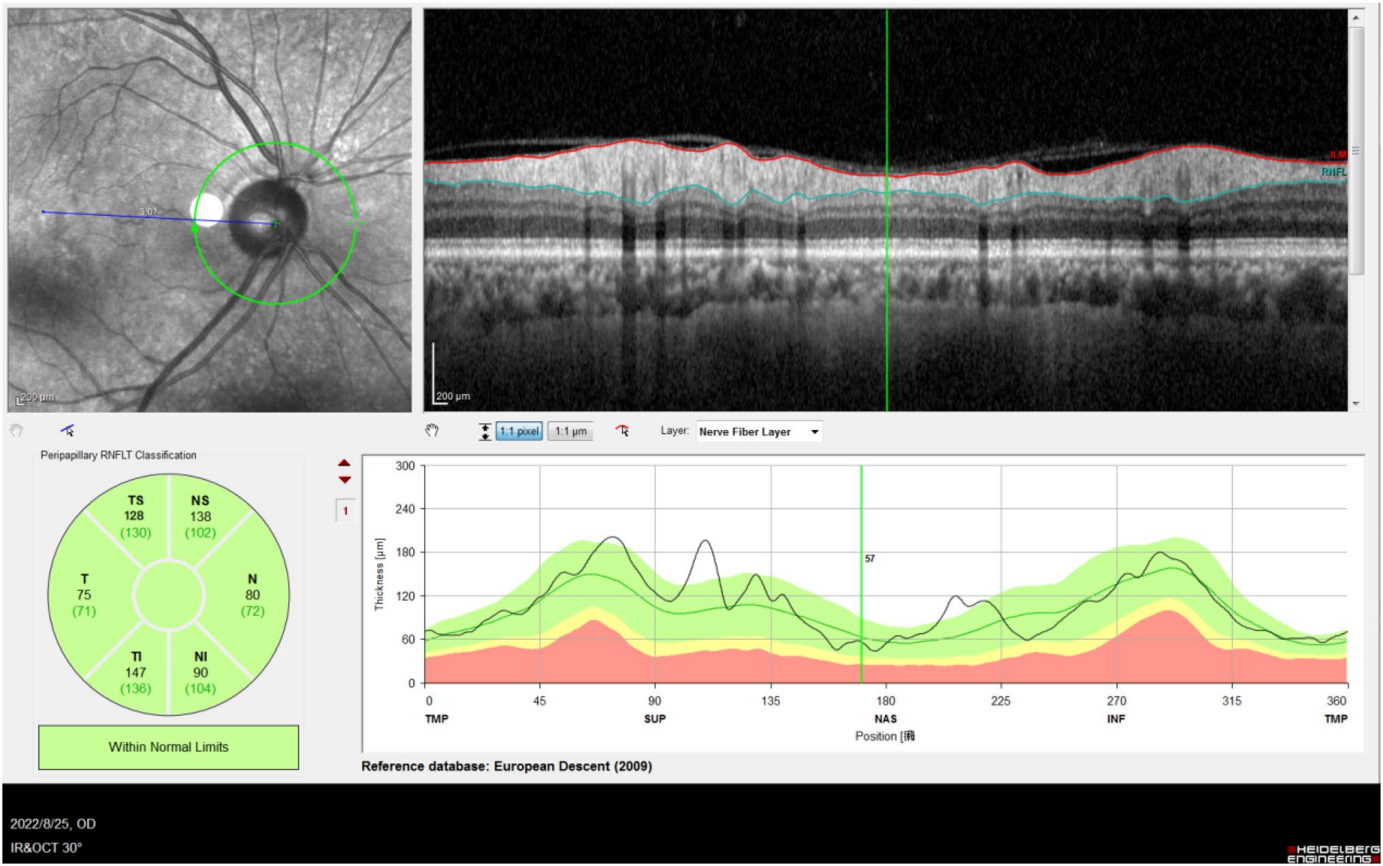
Circumferential scan of the peripapillary retinal nerve fiber layer. The illustration shows an OCT scan of the peripapillary retinal nerve fiber layer of the right eye of a patient. The average thickness of the supranasal (NS), nasal (N), inferonasal (NI), superotemporal (TS), temporal (T) and inferotemporal (TI) subdivisions were recorded on the figure

Using OCT mode with a light source at 870 nm, the peripapillary retinal nerve fiber layer (pRNFL) was measured within 3.7 mm of the peripapillary area around the optic disk. A circular scan with a diameter of 3.7 mm and a depth of 25 mm was employed with the software that comes with the device. For the average thickness, the software is automatically divided into six sections, namely supranasal (Superonasal, NS), nasal (Nasal, N), inferior nasal (Inferonasal, NI), superior temporal (Superotemporal, TS), lateral temporal (Temporal, T), and inferior temporal (Inferotemporal, TI). The mean thickness of each part of the pRNFL was recorded. See Fig. 1.

### Average retinal thickness and vascular density measurement in each layer of the macula

**Fig. 2:**
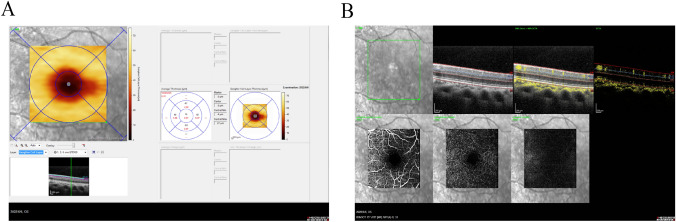
OCTA scans of the macula. Image A shows a 3*3 mm pattern OCTA pattern scan of the right eye of a patient showing the average thickness of the ganglion cell layer in the superior, inferior, nasal, temporal and central subdivisions. Image B documents the superficial vascular complex, deep vascular complex and B-mode images of the superficial vascular complex, deep vascular complex and avascular complex

The OCTA mode was used to record the average thickness of the nerve fiber layer (NFL), ganglion cell layer (GCL), inner plexiform layer (IPL), and inner nuclear layer (INL) in the central region of the retina at 3 × 3 mm, above, below, nasal, temporal and central, using the central macular recess as the center of the scan, with a 3 mm × 3 mm (10°*10°) transverse resolution of 5.7 µm/pixel (512A scan * 512B scan); cell layer (GCL), inner plexiform layer (IPL) and inner nuclear layer (INL). Vascular images of the superficial vascular complex, deep vascular complex and avascular complex were also preserved in 3 × 3 mm OCTA mode scans. Data collection criteria: complete data without loss in the OCTA scan window, uniform color density, no signal interference, OCTA quality intensity ≥ 30. See Fig. 2.

### Terminology

Superficial vascular complex (SVC): inner limiting membrane (ILM) to 10 μm above the IPL.

Deep vascular complex (DVC): extends from 10 μm above the IPL to 10 μm below the outer plexiform layer (OPL).

Avascular complex (AC): 10 μm extension below the OPL to the Bruch membrane.

VD represents the ratio of blood flow area to total area scanned using OCTA.

### Statistical analysis

All statistical analyses were performed using the statistical software SPSS version 25.0. Continuous variables are expressed as the mean ± standard deviation ($\overline{x}\pm \mathrm{S }$), and categorical variables are summarized as frequencies and percentages. Vessel density was analyzed using ImageJ software. Baseline characteristics of the different patient groups were summarized and compared according to the distribution of each variable using Fisher's exact test and t test. Between-group differences in retinal thickness and vessel density were compared using multiple linear regression or the covariance method corrected for the variable of age. Pearson correlation analysis was used to analyze the two correlations between baseline data and retinal thickness and vessel density. Because age was considered to significantly differ between the two groups, we verified the correlation between retinal thickness and vessel density by correcting for the variable of age using partial correlation. The presence or absence of diabetes mellitus was used as a dichotomous variable grouping to perform univariate and multivariate logistic regression analyses to screen for possible correlates of diabetic retinal damage. The variables that entered the model and the predicted probability PRE_1 of the new variables outputting the joint diagnosis when the logistic regression model was established in this study were used to plot the receiver operator characteristic (ROC) curve to determine the joint diagnostic index of diabetic retinal damage. The duration of disease among diabetic patients was grouped as dichotomous variables within 5 years and more than 5 years, and univariate and multivariate logistic regression analyses were performed to screen for retinal damage factors associated with the duration of diabetes. The variables entered into the model and the predicted probability PRE_2 of the new variables outputting the joint diagnosis when the logistic regression model was established in this study were used to plot the ROC curve to determine the joint diagnostic index of retinal damage factors associated with the duration of diabetes mellitus. All statistical analyses were defined as statistically significant at *P* < 0.05.

## Results

### Demographic and clinical data

Ninety-six eyes of 48 diabetic patients and 70 eyes of 35 healthy individuals were evaluated in this study. After rigorous screening, the final OCT thickness examination of the pRNFL included both eyes of all examinees. The OCTA examination of the macular area randomly selected a single eye of each examinee, excluding those of substandard quality, and ultimately evaluated 47 eyes of 47 diabetic patients and 34 eyes of 34 healthy individuals. The mean age of the diabetic patients was 57.58 ± 8.19 years; 17 patients were male and 31 patients were female; all DM patients were DR-free; all DM patients had a mean disease of 8.13 ± 5.73 years and a mean maximum fasting glucose of 13.27 ± 4.12 mmol/L. The mean age of the healthy individuals was 51.14 ± 14.13 years; 14 cases were male, and 21 cases were female. The demographic and clinical characteristics of all study participants are summarized in Table 1. There were no significant differences between the groups in terms of sex, BCVA, or IOP (*p* < 0.05). Healthy individuals and diabetic patients showed significant differences in terms of age (*p* = 0.019).

**Table 1:** Baseline characteristics of the study participants

Parameters	Control	NDR	*p* value
No. of subjects	35	48	NA
No. of eyes	70	96	NA
Gander, male/female	14/21	17/31	0.819^†^
Age, year	51.14 ± 14.13	57.58 ± 8.19	**0.019**^**‡**^
BCVA, logMAR (-0.2/-0.1/0/0.1/0.2)	3/9/52/5/1	2/19/62/11/2	0.542^†^
IOP, mmHg	14.82 ± 2.36	14.84 ± 2.33	0.965^‡^
DM duration, year	NA	8.13 ± 5.73	NA
Max. fasting blood glucose, mmol/L	NA	13.27 ± 4.12	NA

Bold *p* value indicates a statistically significant difference

*NA* not applicable, *NDR* patients with diabetes without clinically detectable retinopathy, *DM* diabetes mellitus, *BCVA* best-corrected visual acuity, *IOP* intraocular pressure, *logMAR* logarithm of the minimal angle of resolution, † Fisher’s exact test, ‡ *t* test between groups

### Comparison of pRNFL thickness, VD, and average retinal thickness in the macular area

Statistical analysis of variance showed that after correcting for age as a baseline variable, the pRNFL thickness was significantly lower in the three subdivisions of TI, NS, and NI in the diabetic group than in normal subjects (*p* < 0.05, see Table 2). In contrast, the NFL-Inferior, NFL-Center, GCL-Superior, GCL-Temporal, and GCL-Inferior showed significantly lower retinal thickness in the diabetic group than in the normal group in all layers of the macula (*p* < 0.05, see Table 3). The vascular density in the macular region, whether in the superficial vascular complex, deep vascular complex or avascular complex, was significantly lower in the diabetic group than in the normal group (*p* < 0.05, see Table 3).

**Table 2:** Comparison of pRNFL average thickness between groups ($\overline{x}\pm \mathrm{S }$)

Parameters	Control (eyes = 70)	NDR (eyes = 96)	*p* value
TS	141.47 ± 10.00	140.28 ± 7.84	0.535
T	83.51 ± 7.04	82.59 ± 7.61	0.401
TI	155.30 ± 11.37	141.05 ± 11.56	**0.000**
NS	120.47 ± 11.86	112.59 ± 13.65	**0.019**
N	74.41 ± 7.21	68.99 ± 12.75	0.061
NI	115.96 ± 15.29	105.22 ± 13.85	**0.008**

Bold *p* value indicates a statistically significant difference. The statistical results in the table corrected for the variable of age

*pRNFL* peripapillary retinal nerve fiber layer, *NDR* patients with diabetes without clinically detectable retinopathy, *TS* Superotemporal, *T* Temporal, *TI* Inferotemporal, *NS* Superonasal, *N* Nasal, *NI* Inferonasal

**Table 3:** Comparison of the vascular density around macula and the average thickness of retinal layers under 3 mm × 3 mm scanning between groups ($\overline{x}\pm \mathrm{S }$)

Parameters		Control (eyes = 34)	NDR (eyes = 47)	*P* value
*Retinal thickness in macular area*				
NFL	Superior	21.94 ± 2.16	21.51 ± 2.33	0.654
	Nasal	20.12 ± 1.32	19.70 ± 2.05	0.405
	Temporal	17.44 ± 1.05	17.53 ± 1.28	0.560
	Inferior	25.35 ± 2.33	23.62 ± 2.91	**0.009**
	Center	10.97 ± 1.75	10.13 ± 1.65	**0.029**
GCL	Superior	51.32 ± 4.57	47.94 ± 4.76	**0.007**
	Nasal	50.09 ± 3.71	47.89 ± 4.65	0.075
	Temporal	47.29 ± 4.60	45.32 ± 3.91	**0.043**
	Inferior	52.29 ± 4.04	49.36 ± 4.73	**0.012**
	Center	13.91 ± 2.72	12.85 ± 2.98	0.127
IPL	Superior	40.50 ± 3.04	40.45 ± 2.89	0.796
	Nasal	41.18 ± 2.82	41.09 ± 2.80	0.884
	Temporal	39.53 ± 3.22	39.68 ± 2.56	0.989
	Inferior	40.97 ± 2.93	40.49 ± 2.81	0.649
	Center	17.24 ± 2.51	17.09 ± 2.17	0.718
INL	Superior	41.62 ± 3.55	42.43 ± 3.51	0.629
	Nasal	40.82 ± 3.32	41.49 ± 4.20	0.844
	Temporal	37.94 ± 2.60	38.77 ± 2.67	0.300
	Inferior	41.26 ± 2.11	41.83 ± 2.71	0.434
	Center	17.76 ± 3.64	18.66 ± 3.82	0.519
*Vascular density *(%)				
SVC		32.05 ± 6.34	27.38 ± 5.74	**0.006**
DVC		24.47 ± 5.32	20.67 ± 6.00	**0.014**
AC		2.8 ± 1.39	2.15 ± 0.93	**0.034**

Bold *p* value indicates a statistically significant difference. The statistical results in the table corrected for the variable of age

*NFL* nerve fiber layer, *GCL* ganglion cell layers, *IPL* inner plexiform layer, *INL* inner nuclear layer, *NDR* patients with diabetes without clinically detectable retinopathy, *SVC* superficial vascular complex, *DVC* deep vascular complex, *AC* avascular complex

### Correlation of pRNFL thickness or VD with basic clinical data

Correlation analysis of pRNFL thickness with baseline data showed that pRNFL-NI thickness was significantly negatively correlated with age (*r* = − 0.279, *p* = 0.011); pRNFL-TI, pRNFL-N, and pRNFL-NI thickness were significantly negatively correlated with DM duration (*r* = − 0.595, *p* = 0.000; *r* = − 0.615, *p* = 0.000 and *r* = − 0.555, *p* = 0.000); see Table 4. Correlation analysis of vessel density with baseline data showed a significant negative correlation between SVC-VD and age (*r* = − 0.299, *p* = 0.007); SVC-VD, DVC-VD, and duration of DM also showed a significant negative correlation (*r* = − 0.578, *p* = 0.000 and *r* = − 0.440, *p* = 0.003); see Table 5.

**Table 4:** Correlation between mean thickness of pRNFL and basic clinical data

Parameters	TS		T		TI		NS		N		NI	
	*r*	*p*	*r*	*p*	*r*	*p*	*r*	*p*	*r*	*p*	*r*	*p*
Age	− 0.002	0.984	0.098	0.376	− 0.208	0.059	− 0.169	0.126	− 0.170	0.125	** − 0.279**	**0.011**
BCVA	0.001	0.994	− 0.023	0.771	0.049	0.528	0.075	0.336	0.058	0.457	0.048	0.536
IOP	− 0.141	0.069	− 0.049	0.529	− 0.009	0.912	− 0.015	0.853	− 0.010	0.895	0.038	0.627
DM duration	− 0.107	0.470	0.095	0.521	** − 0.595**	**0.000**	− 0.249	0.088	** − 0.615**	**0.000**	** − 0.555**	**0.000**
Max. fasting blood glucose	0.252	0.084	0.029	0.842	− 0.065	0.659	0.277	0.056	0.173	0.239	0.075	0.611

Bold *p* values indicate a strong correlation between the two groups, *p* < 0.05

*DM* diabetes mellitus, *BCVA* best-corrected visual acuity, *IOP* intraocular pressure, *TS* Superotemporal, *T* Temporal, *TI* Inferotemporal, *NS* Superonasal, *N* Nasal, *NI* Inferonasal

**Table 5:** Correlation between vascular density and basic clinical data

Parameters	SVC-VD		DVC-VD		AC-VD	
	*r*	*p*	*r*	*p*	*r*	*p*
Age	** − 0.299**	**0.007**	− 0.213	0.056	− 0.245	0.027
BCVA	0.082	0.299	0.037	0.642	− 0.043	0.587
IOP	− 0.030	0.707	− 0.032	0.685	− 0.008	0.924
DM duration	** − 0.578**	**0.000**	** − 0.440**	**0.003**	− 0.282	0.055
Max. fasting blood glucose	− 0.228	0.123	− 0.029	0.844	0.016	0.915

Bold *p* values indicate a strong correlation between the two groups, *p* < 0.05

*SVC* superficial vascular complex, *DVC* deep vascular complex, *AC* avascular complex, *DM* diabetes mellitus, *BCVA* best-corrected visual acuity, *IOP* intraocular pressure, *VD* vascular density

### Correlation of macular VD with the mean thickness of each retinal layer or the duration of DM

Correlation analysis after correcting for age variables showed that most of the NFL and the subdivisional thickness of the GCL in the macula showed a positive correlation trend with vascular density, and IPL-Superior thickness showed the same positive correlation trend with SVC-VD. NFL-Superior, NFL-Nasal, NFL-Inferior, and NFL-Center thicknesses showed a negative correlation with the duration of DM, GCL-Inferior and GCL-Center thicknesses showed a negative correlation with the duration of DM, and IPL-Superior thickness showed a negative correlation with the duration of DM. Superior thickness showed a negative correlation with the duration of DM. However, a positive trend was observed between IPL-Center, INL-Temporal, and INL-Inferior thicknesses and the duration of DM. All statistics are presented in Table 6.

**Table 6:** The correlation between the mean thickness of retinal layers in macular region and blood vessel density and DM duration

Parameters	SVC-VD		DVC-VD		AC-VD		DM duration	
	*r*	*p*	*r*	*p*	*r*	*p*	*r*	*p*
*NFL*								
Superior	0.232	0.121	0.075	0.620	0.099	0.512	** − 0.497**	**0.000**
Nasal	**0.305**	**0.040**	0.123	0.415	0.202	0.178	** − 0.528**	**0.000**
Temporal	0.118	0.436	− 0.132	0.381	0.255	0.088	− 0.084	0.577
Inferior	0.230	0.125	0.173	0.150	0.279	0.060	** − 0.333**	**0.024**
Center	**0.347**	**0.018**	**0.348**	**0.018**	**0.444**	**0.002**	** − 0.510**	**0.000**
*GCL*								
Superior	0.011	0.941	0.227	0.130	0.222	0.138	− 0.122	0.420
Nasal	0.113	0.454	0.257	0.085	**0.307**	**0.038**	− 0.255	0.088
Temporal	0.091	0.548	0.042	0.783	0.185	0.321	0.110	0.467
Inferior	0.271	0.069	0.278	0.061	0.147	0.329	** − 0.404**	**0.005**
Center	0.196	0.192	**0.445**	**0.002**	**0.310**	**0.036**	** − 0.428**	**0.003**
*IPL*								
Superior	**0.354**	**0.016**	0.193	0.199	0.165	0.272	** − 0.307**	**0.038**
Nasal	0.235	0.115	0.184	0.221	0.243	0.103	− 0.112	0.457
Temporal	0.225	0.133	0.067	0.660	0.043	0.774	− 0.236	0.114
Inferior	0.037	0.806	0.064	0.672	0.186	0.217	− 0.088	0.560
Center	− 0.187	0.212	− 0.110	0.467	− 0.116	0.442	**0.443**	**0.002**
*INL*								
Superior	0.107	0.480	0.189	0.208	0.042	0.783	0.112	0.459
Nasal	0.266	0.074	0.288	0.052	0.226	0.131	0.129	0.394
Temporal	− 0.258	0.084	** − 0.316**	**0.033**	0.045	0.765	**0.360**	**0.014**
Inferior	− 0.073	0.632	− 0.151	0.315	− 0.146	0.334	**0.450**	**0.002**
Center	− 0.038	0.802	− 0.068	0.651	0.062	0.682	0.113	0.453

Bold *p* values indicate a correlation between the two groups, *p* < 0.05. The statistical results in the table corrected for the variable of age. *SVC* superficial vascular complex, *DVC* deep vascular complex, *AC* avascular complex, *DM* diabetes mellitus, *NFL* nerve fiber layer, *GCL* ganglion cell layers, *IPL* inner plexiform layer, *INL* inner nuclear layer; *VD* vascular density

### Indicators associated with diabetic retinal damage

Age variables with statistically significant differences in the analysis of baseline correlates were included in the subsequent regression analysis with all OCT and OCTA results. Logistic regression analysis was performed according to the presence or absence of diabetes mellitus; after selecting variables with significant differences for univariate regression analysis, those with significantly different univariate variables were then subjected to multiple regression analysis using the forward step method, and the results of the predictive model parameter estimation and testing are shown in Table 7. The 2 independent variables of retinal thickness included in the model (pRNFL-TI, GCL- Superior) were statistically significant (*p* < 0.05), and the resulting prediction model was tested by the Hosmer‒Lemeshow goodness-of-fit test *χ*2 = 29.607, *p* = 0.000, -2 log likelihood = 80.587, and the prediction accuracy of the model was 74.1%. The logistic regression equation can be obtained as Logit (*p*) = -22.618 + 0.086 × pRNFL-TI + 0.190 × GCL-Superior.

**Table 7:** logistic regression analysis to determine the factors associated with the risk of DR occurrence

Factors	Univariate analyses			Multivariate analyses		
	*B*	OR (95% CI)	*p*	*B*	OR (95% CI)	*p*
Age	− 0.055	0.947 (0.905–0.990)	0.017			
Vascular density (%)						
SVC	0.132	1.141 (1.049–1.246)	0.002			
DVC	0.110	1.124 (1.033–1.223)	0.007			
AC	0.574	1.775(1.111–2.836)	0.016			
Mean thickness of pRNFL						
TI	0.082	1.085(1.040–1.132)	0.000	0.086	1.090(1.041–1.142)	0.000
NS	0.048	1.050(1.011–1.089)	0.010			
N	0.049	1.051(1.005–1.099)	0.031			
NI	0.053	1.054(1.018–1.091)	0.003			
Retinal thickness in macular area						
NFL-Inferior	0.253	1.288(1.066–1.556)	0.009			
NFL-Center	0.298	1.347(1.023–1.772)	0.034			
GCL-Superior	0.163	1.177(1.054–1.315)	0.004	0.190	1.209(1.064–1.374)	0.004
GCL-Nasal	0.126	1.134(1.011–1.273)	0.032			
GCL-Temporal	0.119	1.126(1.002–1.265)	0.046			
GCL-Inferior	0.158	1.171(1.043–1.314)	0.007			

*OR* odds ratio, 95% *CI* 95% Confidence interval, *SVC* superficial vascular complex, *DVC* deep vascular complex, *AC* avascular complex, *NFL* nerve fiber layer, *GCL* ganglion cell layers, *TS* Superotemporal, *T* Temporal, *TI* Inferotemporal, *NS* Superonasal

**Fig. 3:**
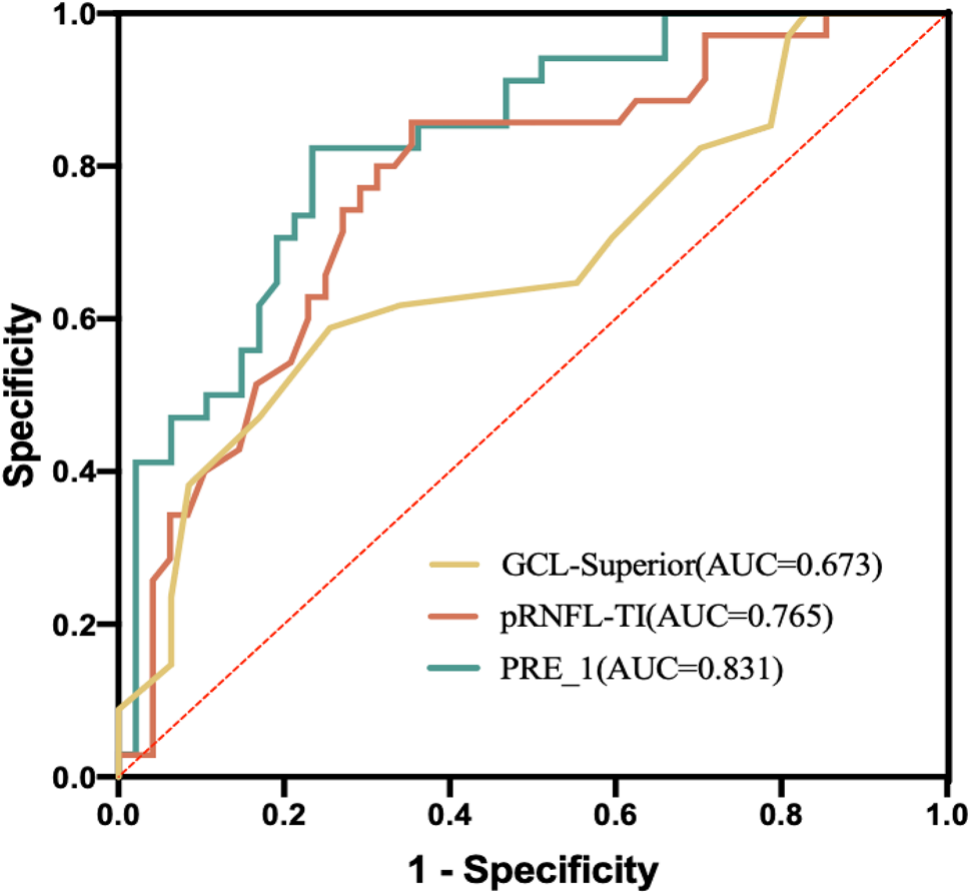
AUCs of retinal OCT and OCTA distinguishing the presence or absence of diabetes mellitus

The ROC curves were plotted by combining the 2 variables that entered the model (pRNFL-TI, GCL-Superior thickness) and the predicted probability of the new variable PRE_1 of the joint diagnosis that was output when the logistic regression model was established in this study, as shown in Fig. 3. The area under the curve was 0.765, 0.673, and 0.831, respectively.

### Indicators of retinal damage related to the duration of diabetes

The continuous variable DM duration was transformed into a dichotomous variable according to the duration of diabetes mellitus within 5 years and more than 5 years, DM duration ≤ 5 years = 0, DM duration > 5 years = 1; variables with significant correlation with the duration of diabetes mellitus in Age, Tables 4, 5, 6 were selected for univariate regression After logistic analysis, the respective significance was collected, and the univariate variables that were significantly different were then subjected to multiple regression analysis using the forward step method, and the results of the prediction model parameter estimation and testing are shown in Table 8. The 2 independent variables (DVC-VD and pRNFL-N thickness) incorporated in the model were statistically significant (*p* < 0.05), and the resulting prediction model was validated by the Hosmer- Lemeshow goodness-of-fit test χ2 = 33.320, *p* = 0.000, -2 log likelihood = 31.645, and the prediction accuracy of the model was 85.1%. The logistic regression equation can be obtained as Logit (*p*) = − 20.294 + 0.296 × DVC-VD + 0.196 × pRNFL-N.

**Table 8:** Logistic regression analysis to identify retinal damage factors associated with time to diabetes

Factors	Univariate analyses			Multivariate analyses		
	B	OR (95% CI)	*p*	B	OR (95% CI)	*p*
Age	0.003	1.003(0.936–1.076)	0.927			
Vascular density (%)						
SVC	0.274	1.315(1.107–1.563)	**0.002**			
DVC	0.194	1.214(1.062–1.388)	**0.005**	0.296	1.344(1.088–1.661)	**0.006**
Mean thickness of pRNFL						
TI	0.085	1.089(1.029–1.152)	**0.003**			
N	0.135	1.144(1.061–1.234)	**0.000**	0.197	1.217 (1.074–1.380)	**0.002**
NI	0.112	1.119(1.046–1.196)	**0.001**			
Retinal thickness in macular area						
NFL-Superior	0.334	1.396(1.023–1.905)	**0.035**			
NFL-Nasal	0.686	1.987(1.258–3.138)	**0.003**			
NFL-Inferior	0.217	1.242(0.993–1.553)	0.057			
NFL-Center	0.713	2.039(1.273–3.265)	**0.003**			
GCL-Inferior	0.187	1.206(1.030–1.412)	**0.020**			
GCL-Center	0.450	1.568(1.126–2.184)	**0.008**			
IPL-Superior	0.216	1.241(0.976–1.577)	0.078			
IPL-Center	-0.324	0.723(0.533–0.982)	**0.038**			
INL-Temporal	− 0.220	0.802(0.631–1.020)	0.072			
INL-Inferior	− 0.306	0.737(0.566–0.959)	**0.023**			

Bold *p* values indicate a correlation between the two groups, *p* < 0.05

95% CI, 95% Confidence interval, *SVC* superficial vascular complex, *DVC* deep vascular complex, *NFL* nerve fiber layer, *GCL* ganglion cell layers, *IPL* inner plexiform layer, *INL* inner nuclear layer, *TS* Superotemporal, *T* Temporal, *TI* Inferotemporal

**Fig. 4:**
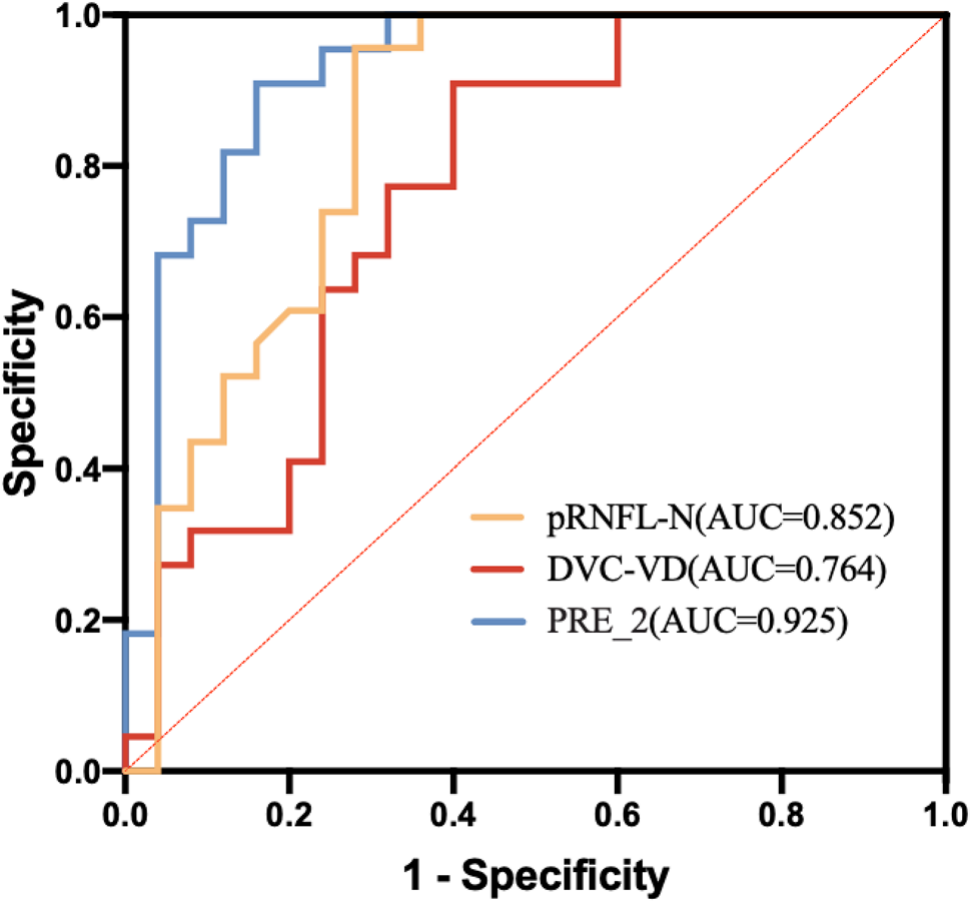
AUCs of retinal OCT and OCTA distinguishing between those with diabetes lasting less than 5 years and those with diabetes lasting more than 5 years

The ROC curves were plotted by taking the 2 variables that entered the model (DVC-VD and pRNFL-N thickness) and the predicted probability of the new variable PRE_2 of the joint diagnosis of the output when the logistic regression model was built in this study (see Fig. 4). The areas under the curves were 0.764, 0.852, and 0.925, respectively.

## Discussion

In this study, we identified several baseline characteristics of DM patients, OCT, and quantitative indicators of their OCAT that may reflect the risk or diagnostic value of developing DR in DM patients. Most studies on early changes in preclinical DR are based on the genetic and protein levels, while clinical changes are unknown [16–18]. We know that the main components of the retinal NVU include neurons, glial cells, and the retinal microvascular system. There are five types of retinal neurons: photoreceptors, horizontal cells, bipolar cells, amacrine cells, and ganglion cells. Photoreceptors are mainly located in the outer nuclear layer (ONL). The cell bodies of horizontal, bipolar, and anaplastic cells are mainly located in the INL. Retinal ganglion cells (RGCs) are distributed in the GCL [19]. The glial component consists of macroglia (astrocytes and Müller cells), microglia, and oligodendrocytes, which are the interface between neurons and the vascular system, regulate functional communication between them and are present between the inner boundary membrane (ILM) and the outer membrane (OLM) [20]. However, retinal microvessels are distributed from the NFL to the INL and are not present in the ONL. In this study, we looked at the functional impairment of the retinal NVU in DM patients, so we investigated the quantitative analysis of OCT and OCTA from the retinal NFL to the INL [8, 21].

An important finding of this study was that retinal VD, NFL, and GCL thickness were lower in DM patients than in healthy subjects, even in the absence of DR (Tables 2 and 3). Correlation analysis of baseline information with retinal indicators revealed (Tables 4, 5, 6) that there was no significant correlation between retinal VD and maximum fasting glucose, although blood glucose level was the most important indicator for the diagnosis of diabetes. pRNFL-NI thickness was significantly and negatively correlated with age, and pRNFL-TI, pRNFL-N, and pRNFL- NI thickness was significantly and negatively correlated with the duration of DM disease. SVC-VD showed a significant negative correlation with age, and similarly, SVC-VD and DVC-VD showed a significant negative correlation with the duration of DM. The macula also showed a negative trend of correlation between the thickness of some NFL subdivisions and the duration of DM. This suggests that age may be a risk factor for the development of DR, and the older the age, the greater the risk of retinal vascular damage. Similarly, the duration of DM disease is also a risk factor for the development of DR, and the longer the duration of DM disease is, the greater the risk of nerve fiber and vascular damage in the retina. This finding is consistent with some previous studies [22]. Our updated study showed a negative correlation between the thickness of the inferior and center subdivisions of the GCL and the duration of DM disease, suggesting that RGCs are damaged as the duration of DM disease increases. The rat model of DR by Wang QC et al. similarly demonstrated that hyperglycemia induces the death of RGCs [23]. To our surprise, INL-temporal and INL-inferior thicknesses showed a positive trend in correlation with the course of DM. We retrieved two articles showing increased IPL and INL thickness in patients with nonproliferative DR (NPDR) compared to those with NDR and attributed this to increased INL thickness due to early microglial activation and aggregation [24]. Bandello F et al. similarly found that the increase in retinal thickness in NPDR patients compared to healthy individuals was located mainly in the INL and extended to the adjacent retinal layers [25]. We know that the INL contains the nuclei of Müller cells and most microglia in addition to the cell bodies of horizontal, bipolar, and anaplastic cells for cell and animal experiments revealed that under high sugar conditions, Müller and microglial cells become active and induce oxidative stress and inflammation [26, 27]. Activation, value-added, edema, and hypertrophy of glial cells in the retinal NVU may be the main causes of INL thickening [28].

In the correlation analysis between retinal thickness and VD in the macula (Table 6), most of the subdivisions of the NFL and GCL in the macula showed a positive trend of correlation with vascular density; however, most of the subdivisions of the INL showed a negative trend of correlation with vascular density. Damage to nerve fibers and RGCs can cause microvascular damage, and the activation, value-added, edema of glial cells, as well as the oxidative stress and inflammation triggered by them, can also lead to alterations in microvascular canal diameter and blood flow. Of course, this effect may also be bidirectional. The physiological demands of the retinal neurons determine the filling of the vessels and the changes in their lumen diameter [29]. The retinal microvasculature provides nutrients and oxygen to the NVU and excretes waste products to meet the high demand for oxygen consumption by the retina, a metabolically active neurovascular tissue [30, 31]. Nerve and glial cell lesions can affect the microvasculature, and microvascular lesions can similarly involve the glia [8].

In the analysis of the predictors of retinal damage in DM, logistic regression analysis was performed according to the presence or absence of DM, and two variables, pRNFL-TI and GCL-Superior thickness, were screened. The AUCs of the 2 variables for the prediction of retinal damage in DM patients differed (0.765 and 0.673, respectively), and the differences between the AUCs of each variable and the predictive ability of the model were statistically significant (*p* < 0.05). The AUC of the model predicting prognosis reached 0.831 when the 2 indicators were combined for diagnosis. According to the judgment criteria of predictive efficacy, AUC > 0.8, the judgment efficacy is excellent, which is more advantageous than applying each variable alone to predict retinal damage in DM patients, suggesting that clinicians can pay attention to the early prevention of retinal damage in DM patients based on the thickness changes in the pRNFL and GCL.

In the analysis of retinal damage indicators associated with the duration of DM disease, the model incorporating DVC-VD and pRNFL-N thickness was statistically significant (*p* < 0.05) after regression logistic analysis according to the duration of DM disease within 5 years and more than 5 years. Combining the two indices for diagnosis, the model predicted an AUC of 0.925, which is excellent for judgmental efficacy and more advantageous than applying each variable alone for predicting retinal damage in patients with long-term DM, suggesting that the duration of DM disease is a nonnegligible factor for DR. Clinicians should thus pay special attention to the nerve fibers and superficial microvessels in the retina of patients with DM over 5 years.

Two of the three indicators in the model analysis with the presence or absence of DM were based on the pRNFL and one on the GCL, and it can be seen that VD did not appear significantly different. However, in the prediction model categorized by 5 years of DM disease, DVC-VD appeared, still with pRNFL thickness. This indicates that the neurological changes around the optic disk may occur earlier in the development of DR, while most of our clinical focus is only on retinal damage in the macula. We should note that the axonal density of the optic papilla is higher than that of the macula. In the early stages, retinal damage may be seen mainly at the neural level, such as RGCs and glial cells. The microvascular changes only appear slowly as the disease continues to extend, which is similar to the findings of Zhang et al. [14]. The process of collecting indicators such as DM disease duration, OCT, and OCTA is relatively simple, and these indicators can be judged in a short period of time, which is suitable to be applied in the disease prognosis assessment of DM patients to indicate the possible course of their disease. Early and effective interventions for patients may have a greater chance at saving visual impairment.

## Conclusion

Chronic hyperglycemia leads to hypoxia and causes retinal inflammation, factors that may contribute to impaired retinal NVU and its coupling mechanism imbalance in diabetic patients. Basic clinical information and rapid noninvasive OCT and OCTA techniques are useful for the quantitative assessment of retinal NVU prognosis in DM patients without retinopathy.

## Abbreviations

DM: Diabetes mellitus
DR: Diabetic retinopathy
NDR: Patients with diabetes without clinically detectable retinopathy
OCT: Optical coherence tomography
OCTA: Optical coherence tomography angiography
NVU: Neurovascular unit
pRNFL: Peripapillary retinal nerve fiber layer
NFL: Nerve fiber layer
GCL: Ganglion cell layers
IPL: Inner plexiform layer
INL: Inner nuclear layer
BCVA: Best-corrected visual acuity
IOP: Intraocular pressure
logMAR: Logarithm of the minimal angle of resolution
SVC: Superficial vascular complex
DVC: Deep vascular complex
AC: Avascular complex
VD: Vascular density
ROC: Receiver operating characteristic
AUC: The area under the ROC
SD: Standard deviation
